# Supplementary figures and images for: The Proteome Landscape of Human Placentas for Monochorionic Twins with Selective Intrauterine Growth Restriction
Source: Genomics Proteomics Bioinformatics. 2023 Apr 29;21(6):1246–59. doi: 10.1016/j.gpb.2023.03.002 (PMC11082409; doi:10.1016/j.gpb.2023.03.002)

A

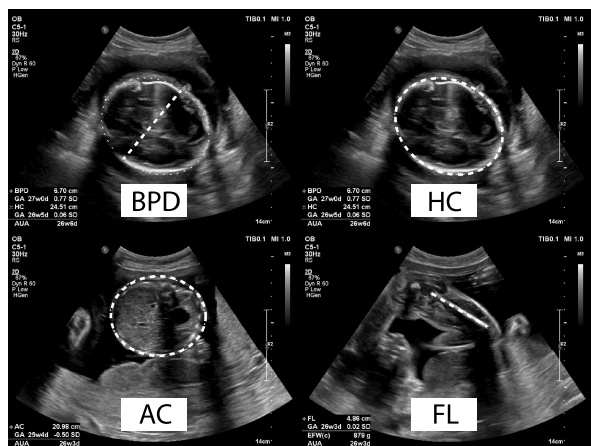

B

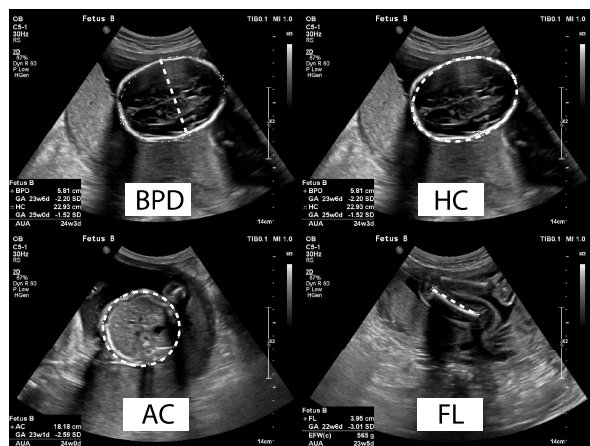

C

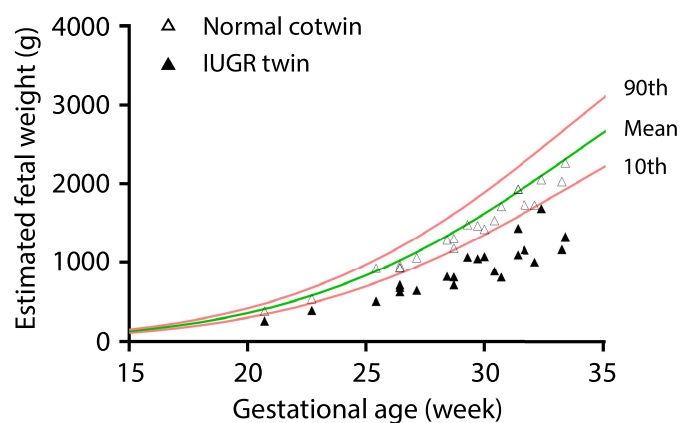

D

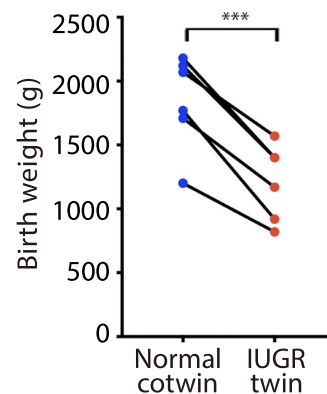

E

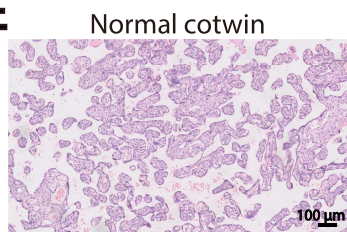

F

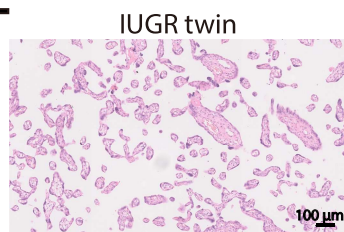

G

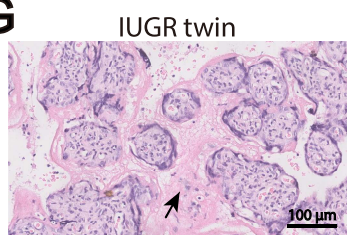

H

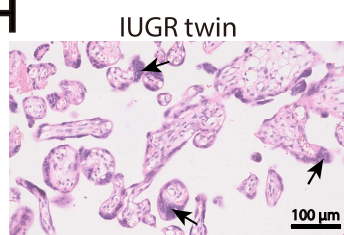

I

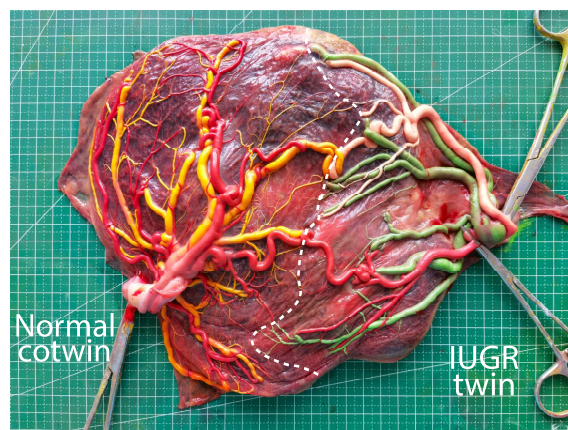

Supplement: Supplementary Figure S1 — Clinical characteristics and placental histopathology of sIUGR twins. Representative images of ultrasound biometric measurements of the normal cotwin (A) and the IUGR twin (B) from a sIUGR pregnancy, including BPD, HC, AC, and FL. C. Ultrasound-estimated fetal weights of the normal cotwins and the IUGR twins from six pairs of sIUGR pregnancies throughout gestation were plotted on the fetal growth curve. D. Birth weight of the normal cotwins and the IUGR twins (n = 6 in each group). E. Normal placental parenchyma from the normal cotwin. Scale bar, 100 µm. F. Distal villous hypoplasia in the IUGR twin placenta. Scale bar, 100 µm. G. Increased fibrin deposition (arrow) in the IUGR twin placenta. Scale bar, 100 µm. H. Increased syncytial knots (arrow) in the IUGR twin placenta. Scale bar, 100 µm. I. A representative image of sIUGR twin placenta after dye injection. Placental sharing is indicated by the white dotted line. Velamentous cord insertion isobserved in the IUGR twin. ***, P < 0.001. IUGR, intrauterine growth restriction; sIUGR, selective IUGR; BPD, biparietal diameter; HC, head circumference; AC, abdominal circumference; FL, femur length. [file mmc1.pdf]

A

## Molecular and cellular functions

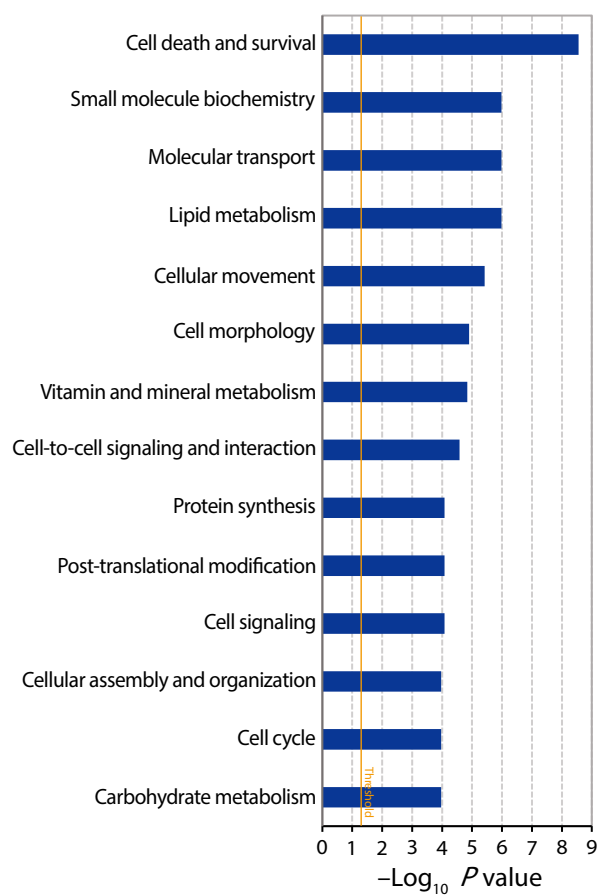

B

## Diseases and disorders

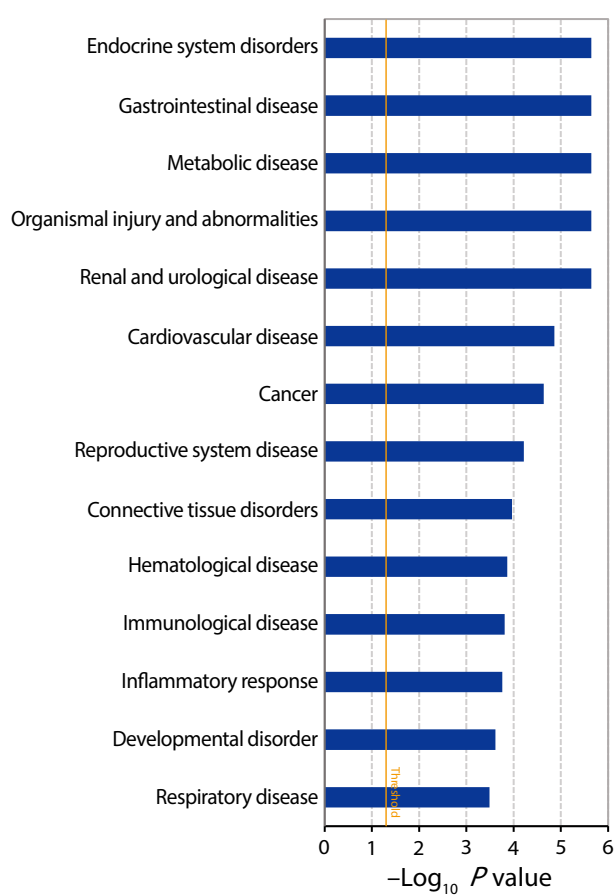

C

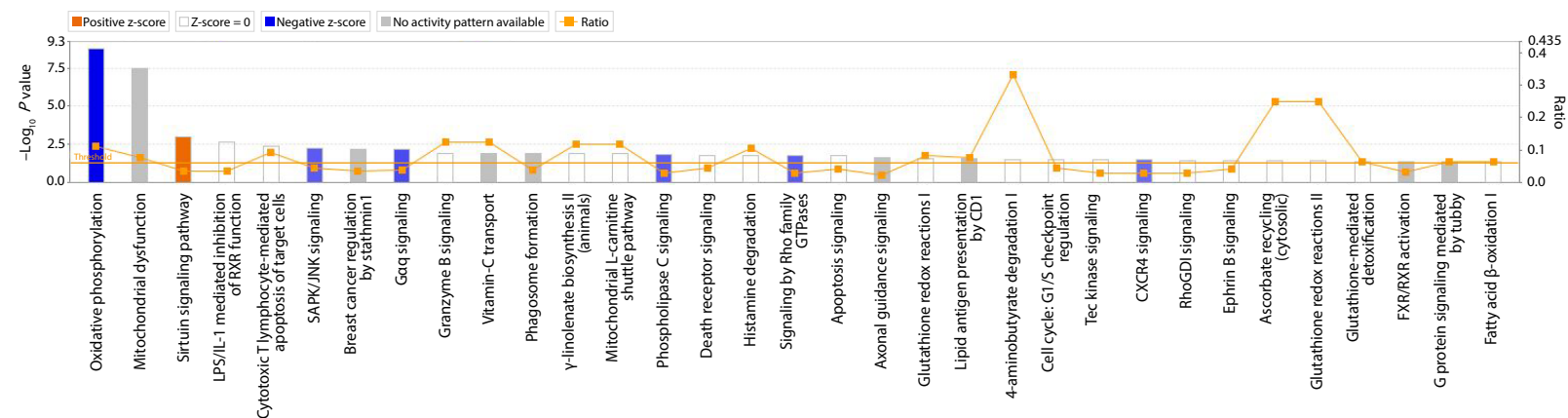

Supplement: Supplementary Figure S2 — Bioinformatic analysis of the DEPs in IUGR twin placentas by IPA software. A. DEPs significantly enriched in the “molecular and cellular function” category. B. DEPs significantly enriched in the “disease and disorder” category. C. Canonical pathway analysis of the DEPs. DEP, differentially expressed proteins; IPA, ingenuity pathway analysis. [file mmc2.pdf]

**A**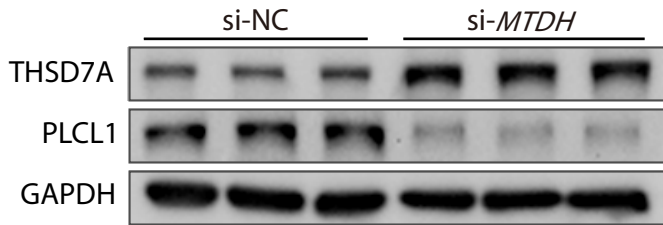**B**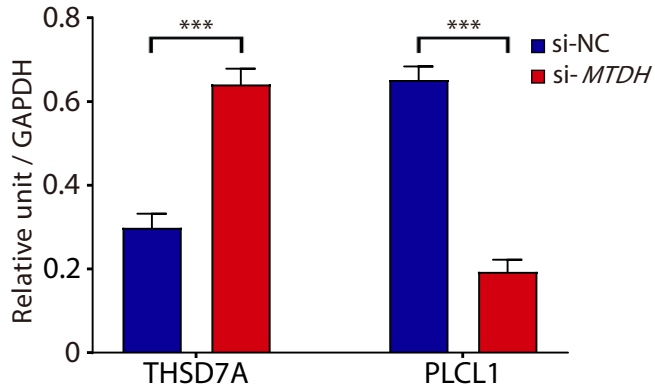

Supplement: Supplementary Figure S3 — MTDH knockdown increases THSD7A protein expression and decreases PLCL1 protein expression in HUVECs. A. Western blotting assay of THSD7A and PLCL1 expression in HUVECs treated with si-NC and si-MTDH. B. The relative intensity of THSD7A and PLCL1 levels were evaluated by ImageJ software. All experiments have 3 replicates, and the results are shown as mean ± SD. ***, P < 0.001. HUVEC, human umbilical vein endothelial cell. [file mmc3.pdf]
