# Supplementary material for: The Proteome Landscape of Human Placentas for Monochorionic Twins with Selective Intrauterine Growth Restriction
Source: Genomics Proteomics Bioinformatics. 2023 Apr 29;21(6):1246–59. doi: 10.1016/j.gpb.2023.03.002 (PMC11082409; doi:10.1016/j.gpb.2023.03.002)
Supplement: Supplementary Table S6 — Primers used for qRT-PCR [file mmc9.docx]

| **Table S6 List of primers used for quantitative real-time PCR** | | |  |
| --- | --- | --- | --- |
| **Gene name** | **Gene description** | **Forward primer sequence (5'–3')** | **Reverse primer sequence (5'–3')** |
| *METAP2* | *Methionyl aminopeptidase 2* | AAAGGACAAGAATGCGAATACCC | CAGGCTTGATCCAGCTCATTAC |
| *EFNB2* | *Ephrin B2* | TATGCAGAACTGCGATTTCCAA | TGGGTATAGTACCAGTCCTTGTC |
| *VIM* | *Vimentin* | GACGCCATCAACACCGAGTT | CTTTGTCGTTGGTTAGCTGGT |
| *MTDH* | *Metadherin* | AAGCAGTGCAAAACAGTTCACG | GCACCTTATCACGTTTACGCT |
| *GAPDH* | *Glyceraldehyde-3-phosphate dehydrogenase* | GGAGCGAGATCCCTCCAAAAT | GGCTGTTGTCATACTTCTCATGG |
